# Analyzing Public Conversations About Heart Disease and Heart Health on Facebook From 2016 to 2021: Retrospective Observational Study Applying Latent Dirichlet Allocation Topic Modeling

**DOI:** 10.2196/40764

**Published:** 2022-11-22

**Authors:** Haoning Xue, Jingwen Zhang, Kenji Sagae, Brian Nishimine, Yoshimi Fukuoka

**Affiliations:** 1 Department of Communication University of California Davis, CA United States; 2 Department of Public Health Sciences University of California, Davis Davis, CA United States; 3 Department of Linguistics University of California Davis, CA United States; 4 Department of Physiological Nursing University of California San Francisco, CA United States

**Keywords:** heart health, heart disease, topic modeling, sentiment analysis, social media, Facebook, COVID-19, women’s heart health

## Abstract

**Background:**

Heart disease continues to be the leading cause of death in men and women in the United States. The COVID-19 pandemic has further led to increases in various long-term cardiovascular complications.

**Objective:**

This study analyzed public conversations related to heart disease and heart health on Facebook in terms of their thematic topics and sentiments. In addition, it provided in-depth analyses of 2 subtopics with important practical implications: heart health for women and heart health during the COVID-19 pandemic.

**Methods:**

We collected 34,885 posts and 51,835 comments spanning from June 2016 to June 2021 that were related to heart disease and health from public Facebook pages and groups. We used latent Dirichlet allocation topic modeling to extract discussion topics illuminating the public’s interests and concerns regarding heart disease and heart health. We also used Linguistic Inquiry and Word Count (Pennebaker Conglomerates, Inc) to identify public sentiments regarding heart health.

**Results:**

We observed an increase in discussions related to heart health on Facebook. Posts and comments increased from 3102 and 3632 in 2016 to 8550 (176% increase) and 14,617 (302% increase) in 2021, respectively. Overall, 35.37% (12,340/34,885) of the posts were created after January 2020, the start of the COVID-19 pandemic. In total, 39.21% (13,677/34,885) of the posts were by nonprofit health organizations. We identified 6 topics in the posts (heart health promotion, personal experiences, risk-reduction education, heart health promotion for women, educational information, and physicians’ live discussion sessions). We identified 6 topics in the comments (personal experiences, survivor stories, risk reduction, religion, medical questions, and appreciation of physicians and information on heart health). During the pandemic (from January 2020 to June 2021), risk reduction was a major topic in both posts and comments. Unverified information on alternative treatments and promotional content was also prevalent. Among all posts, 14.91% (5200/34,885) were specifically about heart health for women centering on local event promotion and distinctive symptoms of heart diseases for women.

**Conclusions:**

Our results tracked the public’s ongoing discussions on heart disease and heart health on one prominent social media platform, Facebook. The public’s discussions and information sharing on heart health increased over time, especially since the start of the COVID-19 pandemic. Various levels of health organizations on Facebook actively promoted heart health information and engaged a large number of users. Facebook presents opportunities for more targeted heart health interventions that can reach and engage diverse populations.

## Introduction

### Background

Heart disease continues to be the leading cause of death in men and women in the United States [1]. In 2020, approximately 690,000 individuals died of heart disease, and heart disease deaths increased by 4.8%, the greatest increase since 2012 [2]. The COVID-19 pandemic may be associated with this significant increase in heart disease mortality because of the disruption of access to health care and treatment [3]. In addition, recent research has documented a variety of long-term cardiovascular complications resulting from COVID-19 [4]. Given the increasing burden of heart diseases, understanding public knowledge and interests in heart disease and heart health is urgently needed to develop public and targeted interventions and communication programs to improve preventive measures and health care access and use for heart diseases in the United States.

### Theoretical Background

Researchers and health care providers have increasingly embraced social media data to understand and engage in public conversations regarding various public health issues, including cardiovascular diseases and heart health. Social media provides a great opportunity to observe and understand the information environment related to heart diseases and health. We based our research inquiries on 2 theoretical backgrounds.

First, we drew on the Health Belief Model, which theorizes how perceived susceptibility, perceived severity, perceived benefits, perceived barriers, cues to action, and self-efficacy work together to influence health behaviors and decisions [5]. Using this theoretical lens, we expect to uncover how social media discussions about heart health reveal the public’s risk perceptions and related theoretical constructs, suggesting important factors to be considered in health communication messages and programs for promoting heart health. Previous research has mostly studied people’s perceptions using self-reported measures [6]. Given the data from social media, we aimed to investigate the presence of the public’s risk perceptions and other related perceptions in this retrospective observational study of social media information exchange.

Second, health-related conversations on social media can affect one’s perceived susceptibility to and severity of heart diseases [7]. Social media discussions can also influence one’s health-related knowledge, with which one may develop a stronger belief in the benefits and effectiveness of preventive behaviors and self-efficacy [8]. It is crucial to construct a high-level overview of heart health–related information on social media to understand the web-based information environment that influences the public’s health beliefs and behaviors [9].

Finally, social media provides a platform for the public to not only obtain access to health information but also connect with each other [10]. The review by Zhang and Centola [11] theorizes social media as a web-based structure that can facilitate various social processes (eg, social support, social comparison, and social influence) for information diffusion and behavior change. Especially relevant to web-based health discussions, social support and collective information exchange can increase efficacy and motivate preventive actions and health behaviors [12]. Understanding web-based exchanges among the public can provide us with more insights into the public’s support dynamics, which can contribute to improved health beliefs and behaviors.

### Study Context and Aims

Facebook is the most popular social media platform worldwide [13]. In 2021, a total of 7 in 10 American adults used Facebook; Facebook had more users than Twitter and Instagram [14]. However, existing studies have only examined Twitter posts and comments regarding cardiovascular disease and its risk factors [15,16]. For instance, Musaev et al [16] studied Twitter conversations related to cardiovascular diseases. They found that only a few state health departments have played a central role in these public conversations, although the topics of these conversations were not specified. Although topic modeling methods have been increasingly used to categorize public opinions on and concerns about certain health topics, there is no comprehensive analysis of the public’s heart health discussions on Facebook, a frequently used social media platform for health concerns. Topic analyses of longitudinal Facebook data can point out gaps in education and intervention efforts and also reveal significant insights into social media use in public engagement with heart health and the population’s knowledge deficit or misbeliefs.

The primary aim of this study was to analyze public Facebook posts and comments related to heart disease and heart health over the past 5 years in the United States. We used Linguistic Inquiry and Word Count (LIWC; Pennebaker Conglomerates, Inc) [17] to analyze the public’s sentiments regarding heart disease and health. We used the latent Dirichlet allocation (LDA) method to extract discussion topics illuminating the public’s interests and concerns regarding heart disease and heart health [18]. Furthermore, we conducted two subgroup analyses by (1) stratifying the data by gender and zooming in on conversations on heart health for women and (2) comparing the conversations before and during the COVID-19 pandemic. The rationale for delving into these 2 issues is as follows. First, cardiovascular disease is a leading cause of death in women, and the number of deaths in women has been exceeding that in men [19]. However, public awareness of women-specific risks, symptoms, and prevention remains low [20]. Identifying the concerns and discussions specifically related to heart health for women can inform better public communication and interventions for women. Second, COVID-19 has exposed people with preexisting cardiovascular conditions to greater risks, coupled with negative health outcomes because of social isolation and decreased physical activity [21,22]. Understanding conversations during the pandemic provides us with valuable information about the real impact of COVID-19 on people with cardiovascular conditions and their concerns, which will help us cope with similar public health emergencies in the future. With this study that aimed to analyze public discussions and communication patterns on heart health and heart disease on Facebook, the findings can provide new insights into the design of effective health communication and intervention programs to reduce the burden of heart disease in the United States.

## Methods

### Retrospective Study Design

In this retrospective observational study, we collected US posts and comments in English related to heart disease and heart health from Facebook using the *CrowdTangle* (Meta Platforms) data monitoring platform [23]. CrowdTangle is a tool from Meta (Facebook’s parent company) that tracks social media conversations and related data. We extracted the data from June 2016 to June 2021 for the cohort of social media users in the United States, tracing the first available heart disease and health–related Facebook data available on CrowdTangle until the end date of data collection.

### Ethics Approval

This study was approved by the University of California, San Francisco Institutional Review Board (21-34235).

### Facebook Data Extraction

#### Identification and Deduplication

**Figure 1:**
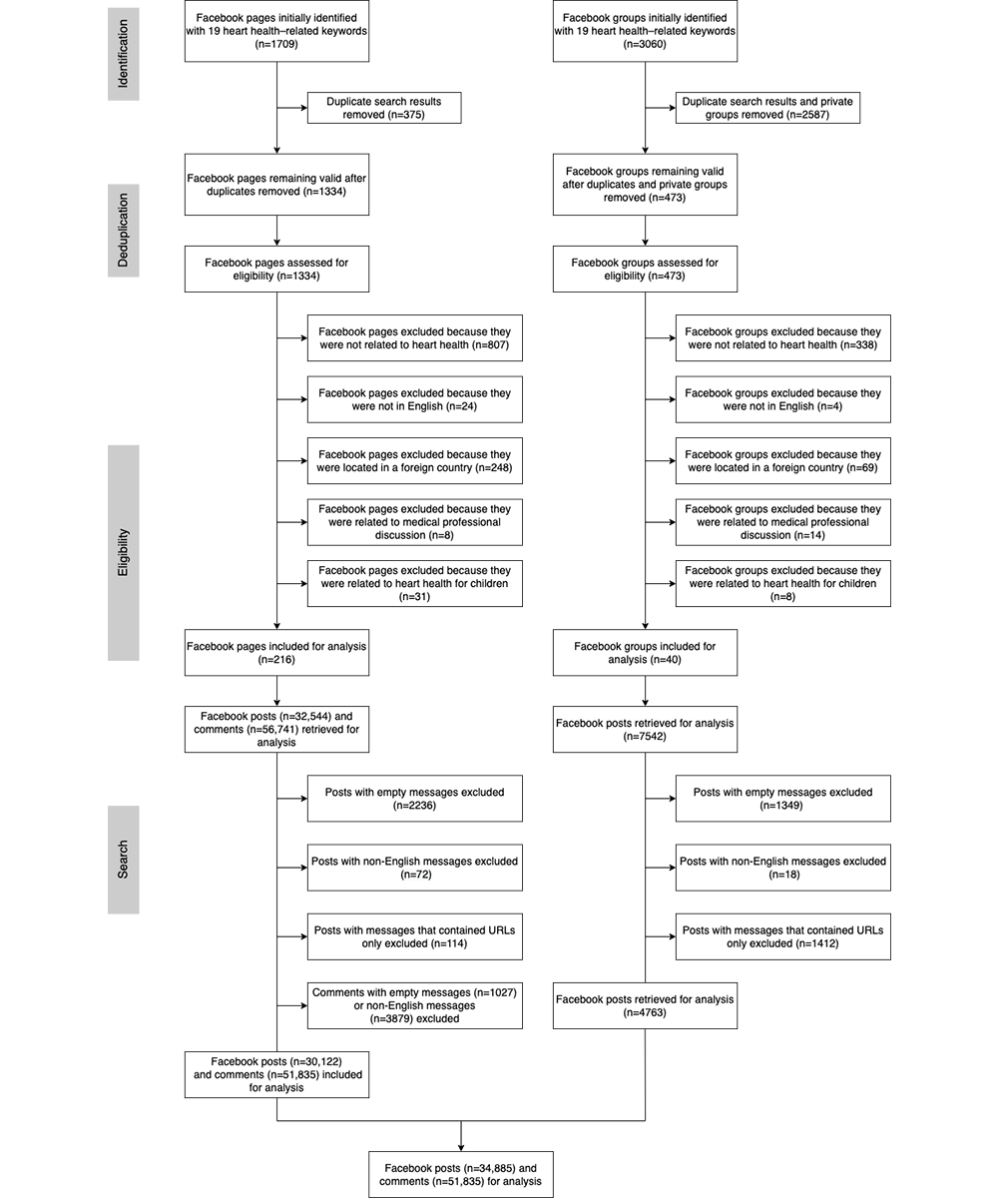
Flow diagram of data extraction and collection processes from public Facebook pages and groups.

Figure 1 shows the data extraction process covering public Facebook pages, groups, posts, and comments. We compiled a set of 19 search keywords related to heart disease (eg, *heart attack*), heart health (eg, *heart health symptoms*), social support (eg, *heart attack support*), and campaigns related to heart health and heart disease (eg, *Go Red for Women*; see Table S1 in Multimedia Appendix 1 for the complete search term list). We searched Facebook pages and groups using the keywords and a web scraping tool, *Selenium* Python [24]. We searched each keyword on Facebook for both public pages and public groups and retrieved all results for each search task. In total, we conducted 38 searches. After retrieving all pages and groups, we removed duplicates and private groups because of no data access, resulting in 1334 pages and 473 groups.

#### Eligibility

As a robustness check, 2 trained research assistants screened for the relevance of the resulting pages and groups. Pages and groups were excluded if they were (1) private or closed (ie, not public), (2) not related to heart disease or heart health, (3) not in English, (4) about pets or animals (eg, animal vaccination), or (5) in a specified foreign location. The 2 research assistants coded a random 10% (100/1334, 7.5% of pages and 48/473, 10.1% of groups) sample of the list. They achieved a 94% agreement rate for page coding and 89% for group coding. Finally, we included 216 public pages and 40 public groups for data collection and analysis.

#### Search

Next, we searched within the Facebook pages and groups and collected posts related to heart health and heart disease using CrowdTangle [23]. We then retrieved public comments attached to all the posts from Facebook pages using Facepager [25] as CrowdTangle does not track comments and Facepager provides access to comments on Facebook pages only. Owing to the restriction of the Facebook Graph application programming interface, we could not access comments to posts from Facebook groups. In addition, we collected data on post metrics such as the number of comments, likes, and shares, as provided by CrowdTangle. After collecting all posts and comments, we conducted additional human checking to ensure that the data were relevant and useful for textual analysis. We excluded posts and comments that (1) contained no text (ie, posts with images, videos, or URLs only) or (2) were not in English. Finally, we obtained 34,885 posts and 51,835 comments for analysis.

### Analytical Strategy

We first used LIWC [17] to obtain the sentiments of the posts and comments and explore public sentiments on heart health. LIWC is a software program that captures linguistic features and sentiments in texts using dictionary-based methods. For example, LIWC calculates positive emotions in a given document by counting the percentage of words that appear in the dictionary indicating positive emotions. It has been widely used to analyze health-related conversations on social media and identify the public’s emotions and attitudes [26].

We then performed topic modeling on the data using LDA [18], a widely used computational approach that discovers thematic topics by identifying the co-occurrence of words in different documents. We ran LDA topic modeling with *Gensim* (RARE Technologies Ltd) in Python for the data set of posts and the data set of comments separately [27]. Each LDA model reported the number of topics identified for a given data set, the top 10 words that contributed to a topic, and their relative weights. The optimal number of topics was determined based on the perplexity score of the LDA model [27]. We also extracted the relative weight of each topic for each post or comment, which was used to identify the most relevant topic a post or comment was associated with. One author and a trained research assistant qualitatively analyzed the prominent keywords and associated texts to develop meaningful topic interpretations.

### Heart Disease and Heart Health for Women

To examine the discussion of heart disease and heart health for women specifically, we delved into posts and comments that were analyzed as belonging to the one special topic on heart health for women from the topic analysis results. This included posts (5200/34,885, 14.91%) and their attached comments (9501/51,835, 18.33%) that received a higher topic weight for the one topic on heart health for women than for all other topics.

### Heart Health Before and During the COVID-19 Pandemic

To discern differences in the discussions before and during the COVID-19 pandemic, we separated the data set into pre–COVID-19 posts and comments (before January 1, 2020; 22,545/34,885, 64.63% of posts and 32,774/51,835, 63.23% of comments) and post–COVID-19 posts and comments (after January 1, 2020; 12,340/34,885, 35.37% of posts and 19,061/51,835, 36.77% of comments). Although the first case of COVID-19 in the United States was confirmed on January 21, 2020 [28], we selected January 1, 2020, as the cutoff date as COVID-19 had already received public attention since December 2019 when it started.

### Statistical Analysis

To analyze and compare the level of emotions in posts and comments, we used 2-tailed 2-sample *t* tests to compare the levels of different emotions within posts and comments [29]. Similarly, we used 2-sample *t* tests to compare the same emotion between posts and comments. Finally, we used 2-sample *t* tests to compare the level of emotions in posts and comments before and during the COVID-19 pandemic. Although the sentiments in posts and comments were nonnormal and left-skewed, it is still robust to use *t* tests given the large sample size in this study [30]. In addition, we performed nonparametric tests (ie, Wilcoxon signed-rank tests) and found consistent results.

## Results

### Descriptive Statistics

**Figure 2:**
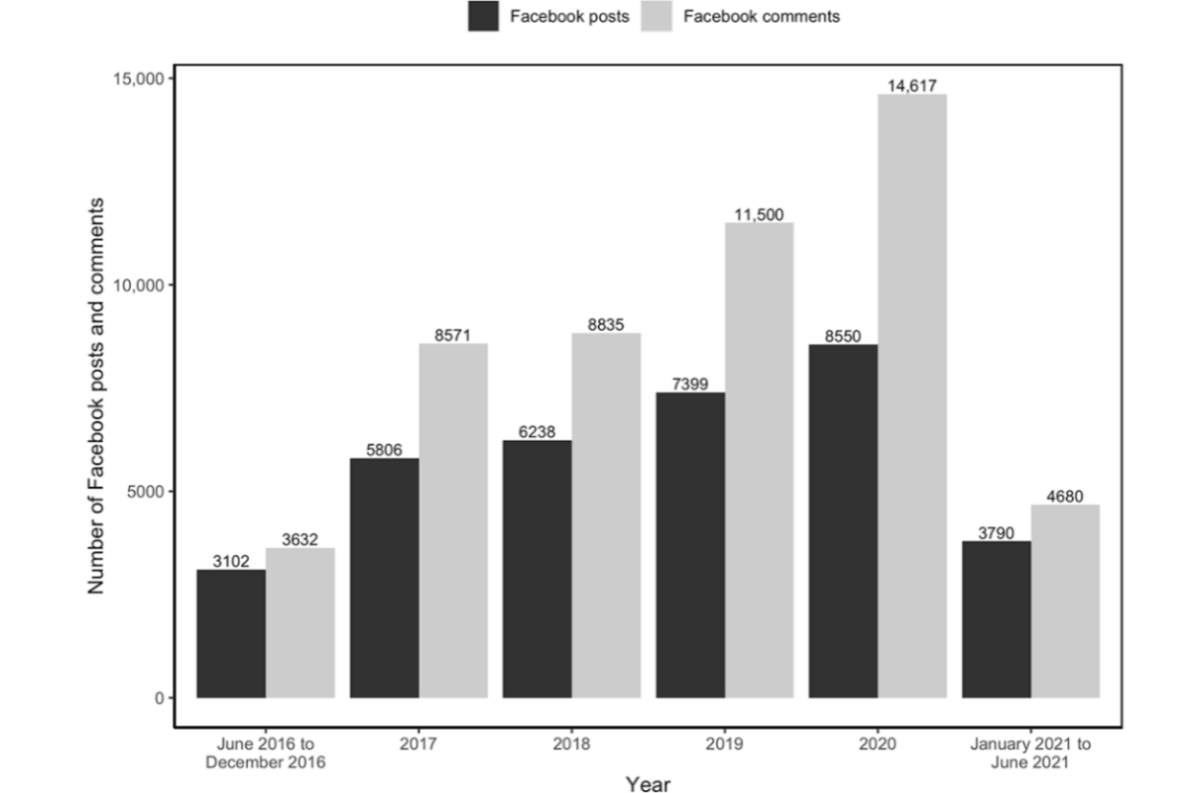
The number of Facebook posts and comments in the United States regarding heart health from June 1, 2016, to June 30, 2021.

We obtained 34,885 Facebook posts and 51,835 comments (attached to 8885 unique posts) for analysis. Figure 2 shows the distribution of the number of posts and comments from June 2016 to June 2021. Both posts and comments increased steadily over the past 5 years. A post on average contained 51.84 (SD 58.93; median 35) words and generated 49.15 (SD 236.31) likes, 4.79 (SD 20.92) comments, and 16.44 (SD 104.59) shares. Comments were significantly shorter than posts, with 17.88 (SD 30.08; median 9) words on average.

### Sentiment in Posts and Comments

We obtained the level of positive and negative emotions with LIWC for posts and comments and used 2-sample *t* tests to compare the level of emotions (Table S2 in Multimedia Appendix 1). In the posts, there were significantly more positive emotions than negative emotions (*P*<.001). Comments also had more positive emotions than negative emotions (*P*<.001). In addition, comments showed significantly more positive emotions and significantly more negative emotions than posts.

Regarding specific negative emotions, LIWC only reported scores for anxiety, anger, and sadness. Posts contained more anxiety (*P*<.001) and anger (*P*<.001) than sadness, whereas comments contained significantly more anger than anxiety (*P*<.001) and sadness (*P*<.001). Overall, both posts and comments contained more positive than negative emotions. Compared with posts, comments were more emotional than posts, with more positive emotions and anger.

### Thematic Topics of All Posts

For the post data set, we extracted 6 thematic topics. Table 1 summarizes the topic keywords and weights, topic interpretations, and example posts for each topic. The topic sequence was determined by the number of posts associated with each topic. Topic 1, heart health promotion, had the greatest number of posts and was about promoting heart health and local events for heart disease and stroke prevention and support provided by national and local organizations. For instance, the American Heart Association has been promoting national campaigns such as Go Red for Women, and state-level organizations of the American Heart Association have promoted localized events such as hiking on their own Facebook pages. Topic 2, sharing personal experiences, included posts that encouraged people to share personal experiences related to heart disease and heart health or posts sharing personal experiences to increase public awareness. Topic 3, risk-reduction education, centered on information related to risk reduction and lifestyle modifications for heart health. Topic 4, heart disease and health promotion for women, contained posts that specifically aimed at promoting heart health for women and emphasized the distinctions in symptoms and warning signs of heart diseases between women and men. Topics 5 and 6 revolved around sharing resources related to heart health. The major difference is that topic 5, educational information sharing, was about heart health–related articles and videos shared by health care professionals in the web-based space, as indicated by the extremely high word counts. In contrast, posts on topic 6, physicians live discussion sessions, promoted live Facebook sessions of physicians and cardiologists sharing heart health–related information.

Table 2 shows the average social media metrics (ie, the number of likes, comments, and shares) from Facebook as well as word count and sentiments from LIWC. Women-specific information on heart health was well liked and considered valuable as posts on topic 4 on average received the most likes and shares of all 6 topics. The public participated and commented the most on posts sharing information and relevant resources (topic 5) and physicians’ live sessions (topic 6). Results from LIWC showed that heart health promotional posts on topic 1 were the most positive, whereas posts concerning risk reduction on topic 3 were mostly negative.

**Table 1:** Latent Dirichlet allocation topic modeling for all Facebook posts, showing topic keywords and weights, topic interpretation, and example posts (N=34,885).

Topic number	Topic name	Top 10 keywords and weights^a^	Interpretation by authors	Example of Facebook posts (paraphrased)
1	Heart health promotion	0.052*heart 0.022*health 0.018*disease 0.013*american 0.012*stroke 0.011*association 0.011*thank 0.011*join 0.010*live 0.009*support	Heart health–, heart disease–, and stroke-related events and support by organizations (eg, the American Heart Association)	The Heart Walk is how the American Heart Association mainly raises funds to prevent heart disease and stroke. It promotes physical activity and healthy heart living, and creates a family-friendly environment. On April 1st, a Saturday, the AHA is holding their annual Franklin County Heart Walk at the Washington City Fairgrounds Swine Pavilion at 9 a.m., with the walking starting at 10.
2	Sharing personal experiences	0.043*heart 0.018*attack 0.009*know 0.009*life 0.008*time 0.008*day 0.008*go 0.007*years 0.007*feel 0.007*family	Sharing personal and family stories related to heart disease and encouraging people to share their stories to increase public awareness	It's been 5 years since I had my heart attack. I waited for about 15 hours with symptoms coming and going before I decided to drive myself to the hospital. After my heart attack, I was traumatized by the fear of death, and I started to exercise and eat healthier. It’s important it is to know the symptoms and listen to your body because one day it could save your life.
3	Risk-reduction education	0.052*heart 0.027*disease 0.024*risk 0.023*blood 0.015*health 0.013*pressure 0.011*high 0.010*stroke 0.009*cholesterol 0.008*study	Risk reduction (eg, blood pressure and cholesterol) and lifestyle modification for heart health and disease and stroke	Eat something healthy and delicious in Barbecued salmon, sauteed zucchini, sweet potatoes, and asparagus. Control your heart health by lowering cholesterol and salt intake.
4	Heart disease and heart health promotion for women	0.053*women 0.048*heart 0.040*red 0.028*disease 0.012*attack 0.010*wear 0.008*available 0.008*symptoms 0.008*awareness 0.007*abstract	Promoting awareness of myocardial infarction symptoms for women and emphasizing characteristics of women’s myocardial infarction by the Go Red for Women Campaign	#GoRedForWomen today. We're bringing attention to women’s heart disease. Women have different warning signs for heart attacks.
5	Educational information sharing	0.050*article 0.047*video 0.042*content 0.035*presentation 0.023*information 0.021*health 0.013*heart 0.012*purpose 0.012*attack 0.012*general	Presenting articles and videos related to heart health and myocardial infarction information	Dr. A, Consulting Physical, discusses heart attack prevention.
6	Physicians’ live discussion sessions	0.065*dr 0.036*heart 0.027*cardiology 0.026*attack 0.023*discuss 0.020*page 0.018*cardiologist 0.018*facebook 0.016*pm 0.014*live	Live Facebook sessions by physicians to discuss myocardial infarctions	Dr. B discussed how to reduce cardiovascular events in a Facebook LIVE session.

^a^The asterisk (*) shows the weight of each keyword.

**Table 2:** Latent Dirichlet allocation topic modeling for all posts, showing the topics’ post distribution, Facebook metrics, and sentiments from Linguistic Inquiry and Word Count (LIWC; N=34,885).

Topic number	Topic name	Posts, n (%)	Facebook metrics^a^				Sentiments from LIWC^b^		
			Number of likes, mean (SD)	Number of comments, mean (SD)	Number of shares, mean (SD)	Word count, mean (SD)		Positive emotion percentage, mean (SD)	Negative emotion percentage, mean (SD)
1	Heart health promotion	10,912 (31.3)	37.67 (264.95)	2.31 (14.51)	8.81 (50.78)	47.64 (36.70)		5.26 (5.16)	1.12 (2.05)
2	Sharing personal experiences	8094 (23.2)	48.37 (205.55)	8.04 (26.47)	15.14 (113.92)	59.02 (77.90)		4.84 (5.58)	3.02 (4.12)
3	Risk-reduction education	8557 (24.5)	49.63 (217.25)	2.82 (17.36)	18.85 (108.32)	43.04 (57.96)		4.87 (8.56)	4.14 (4.54)
4	Heart disease and heart health promotion for women	5200 (14.9)	68.65 (276.00)	5.56 (24.39)	33.36 (166.07)	44.41 (53.66)		2.53 (3.57)	2.62 (4.03)
5	Educational information sharing	1208 (3.5)	63.65 (114.14)	10.43 (18.39)	5.77 (18.25)	137.98 (40.63)		3.02 (7.25)	2.78 (1.37)
6	Physicians’ live discussion sessions	924 (2.6)	58.21 (152.55)	11.97 (32.09)	14.03 (46.10)	49.2 (34.61)		1.38 (2.10)	3.19 (3.00)

^a^Data collected in November 2021.

^b^Positive and negative emotions represent the percentage of words in a post that appear in the dictionary indicating positive and negative emotions.

### Thematic Topics of All Comments

We extracted 6 topics from the comments. These topics centered on personal experience sharing and social interactions. Table 3 lists all topics with keywords and examples. Topic 1, sharing personal experiences, was about sharing one’s experience with heart diseases, physicians, and health insurance. Topic 2, survivor stories, centered on individuals with a history of congenital heart disease sharing their stories when they were young. Social interactions in the comments took the form of discussions, social support, and information sharing. Topic 3, risk-reduction discussion, included comments where people discussed daily risk reduction related to diets, exercise, and smoking for better heart health. Topic 4, religious content, included comments with religious content such as prayers and expressing thanks to God. Topic 5, asking medical questions, revolved around interactions with physicians by asking questions related to heart diseases and risk reduction. Topic 6, sharing appreciation and information, was about people providing social support for each other, appreciating useful information shared by others, and interacting with their social network by tagging their friends in the comments.

Table 4 summarizes the distribution of the 6 topics in the heart health–related comments. Comments to heart health–related posts showed various levels of emotions. Comments on topic 4 had an extremely high level of positive emotions and a low level of negative emotions, suggesting a community with positive and prosocial interactions. In contrast, posts and comments about risk reduction (topic 5) had the most negative emotions.

**Table 3:** Latent Dirichlet allocation topic modeling for all Facebook comments, showing topic keywords and weights, topic interpretation, and example comments (N=51,835).

Topic number	Topic name	Top 10 keywords and weights^a^	Interpretation by authors	Example of Facebook comments (paraphrased)
1	Sharing personal experiences	0.013*go 0.011*heart 0.010*time 0.010*like 0.009*think 0.009*tell 0.009*doctor 0.009*know 0.009*pain 0.009*get	People shared personal stories related to heart disease, physicians, and insurance.	The cardiologist never explained what was going on, and the ER doctors also never said except they needed more tests to make money from you. [I] am afflicted with cardiomyopathy and afib, making my hands and feet cold from poor circulation.
2	Survivor stories	0.065*heart 0.031*years 0.026*surgery 0.022*valve 0.017*ago 0.017*old 0.016*attack 0.014*year 0.013*open 0.012*dr	Survivors shared personal experiences with congenital heart disease when they were young.	Heart Warrior! Had pulmonary valve stenosis, subvalvular stenosis, and artery stenoses all surgically helped in 1993. Another surgery down the line. Fundraised and walked for CHD, grateful for those who also support current and future heart warriors!
3	Risk-reduction discussion	0.042*heart 0.026*disease 0.016*eat 0.016*healthy 0.014*red 0.012*diet 0.012*health 0.011*smoke 0.010*exercise 0.010*risk	Discussion on risk factors and risk reduction to prevent heart disease and improve health (eg, diet, exercise, and smoking cessation)	A healthy lifestyle helps! Water over sweetened beverages and being active keeps the heart healthy! My family has a high BP history, and I need to reduce the sodium in eating, as well as walk more.
4	Religious content	0.061*thank 0.031*god 0.029*good 0.020*bless 0.018*love 0.017*share 0.013*great 0.011*family 0.010*happy 0.010*amaze	Religious content—thanks to God and others	H is beautiful in the pictures, I wish [H] luck. [H] is amazing and kind, Peace with God. It calmed me, and I prayed. I'm doing well after 5 hospital visits, thank you Jesus. Blessed and at home with family.
5	Asking medical questions	0.121*heart 0.097*attack 0.017*congratulations 0.017*women 0.013*symptoms 0.012*sir 0.012*sign 0.008*cause 0.007*patient 0.006*patients	People ask physicians about heart diseases and risk reduction.	What are the precautions for a silent heart attack? Can it be removed? Women’s symptoms are different from mens (not as widely known)
6	Sharing appreciation and information	0.031*great 0.031*love 0.028*information 0.024*nice 0.023*awesome 0.019*sir 0.018*good 0.017*dr 0.011*job 0.011*pressure	People appreciate good information shared by others and organizations and share with their Facebook friends by tagging their names in the comments.	Dr. W, Dr. M; they listened and respected me. Good information in understandable language.

^a^The asterisk (*) shows the weight of each keyword.

**Table 4:** Latent Dirichlet allocation topic modeling for all comments, showing comment distribution, Facebook metrics, and sentiments from Linguistic Inquiry and Word Count (LIWC; N=51,835).

Topic number	Topic name	Facebook metrics^a^—comments, n (%)	Sentiments from LIWC^b^		
			Word count, mean (SD)	Positive emotion percentage, mean (SD)	Negative emotion percentage, mean (SD)
1	Sharing personal experiences	14,000 (27)	33.72 (46.15)	4.29 (11.34)	3.41 (7.40)
2	Survivor stories	7026 (13.6)	18.95 (22.61)	5.93 (13.98)	2.18 (4.49)
3	Risk-reduction discussion	7080 (13.7)	16.39 (25.26)	4.94 (10.93)	3.39 (6.24)
4	Religious content	11,254 (21.7)	10.03 (13.90)	23.44 (22.05)	0.91 (5.12)
5	Asking medical questions	5964 (11.5)	9.33 (9.21)	4.87 (15.11)	6.93 (9.37)
6	Sharing appreciation and information	6511 (12.6)	5.71 (10.08)	23.87 (26.99)	0.89 (4.11)

^a^Data collected in November 2021.

^b^Positive and negative emotions represent the percentage of words in a post that appear in the dictionary indicating positive and negative emotions.

### Thematic Topics of Pre–COVID-19 Posts and Comments

We identified 5 topics for pre–COVID-19 posts and comments (Table S3 in Multimedia Appendix 1 shows topic summaries and examples). The topics identified for pre–COVID-19 posts were similar to the topics identified for all posts: topic 1, promoting experience sharing, was about heart health organizations encouraging the public to share personal experiences; topic 2, sharing local events, centered on the promotion of local events related to heart health; topic 3, risk-reduction discussion, was about risk reduction and lifestyle modification; topic 4, sharing warning signs, was about information related to warning signs and symptoms of specific heart diseases such as hypertrophic cardiomyopathy and cardiac arrest; and topic 5 was about Facebook live sessions of physicians and cardiologists.

Of all topics, topic 4 was the most popular, with the highest number of shares (mean 70.99, SD 264.09) and likes (mean 36.73, SD 174.07), which indicated that people with heart health concerns cared about the warning signs and symptoms of myocardial infarctions (see Table S4 in Multimedia Appendix 1 for summary statistics). Consistently, most negative emotions (mean 4.28, SD 4.59) were expressed when discussing life modifications and risk-reduction methods in topic 3.

In the comments (see Table S5 in Multimedia Appendix 1 for topic summaries and examples), topic 1, sharing warning signs, revolved around people sharing personal experiences related to heart health, including their symptoms and warning signs and diagnoses by different physicians. Topic 2, sharing risk reduction, involved discussions on social relationships that were influenced by heart diseases and their daily risk-reduction practices. Similarly, in topic 3, providing emotional support, people interacted with physicians by expressing appreciation and with others by providing social support and encouragement. Topic 4, religious content, was about religious discussions and appreciation. Topic 5, general health discussions, involved health-related topics other than heart health, such as using e-cigarettes for smoking cessation.

The public expressed the least positive emotions (mean 2.37, SD 4.74) and the most negative emotions (mean 4.01, SD 6.35) in comments on topic 1, where people shared negative emotions, symptoms, and experiences (Table S6 in Multimedia Appendix 1). In contrast, the most positive emotions were expressed on topics 3 (mean 16.64, SD 22.77) and 4 (mean 23.17, SD 27.18), where people were particularly positive in providing emotional support.

### Thematic Topics of Post–COVID-19 Posts and Comments

We discovered 5 topics in post–COVID-19 posts (see Table S7 in Multimedia Appendix 1 for topic summaries and examples). During the pandemic, topic 1 was about physicians’ live discussion sessions. Topic 2 was about general risk-reduction discussions and tips. Topic 3 centered on risk-reduction discussions and awareness promotion specifically for women. Topic 4, risk-reduction discussions for the pandemic, specifically focused on health tips on daily risk reduction during the pandemic. It was more important for people with heart health risks to pay attention to their diet and exercise with stay-at-home orders and social isolation. These posts encouraged people to eat healthily and exercise more at home to maintain a good heart health during the pandemic, which is important for the control and prevention of cardiovascular disease. Topic 5 was about resource sharing related to heart health.

Furthermore, during the pandemic, the public liked (mean 61.55, SD 112.25) and commented (mean 10.08, SD 18.12) on posts related to topic 1 the most (see Table S8 in Multimedia Appendix 1 for detailed statistics). This suggests that the public had a heightened need to seek information and interact with physicians on the web during the pandemic. Live Facebook discussion sessions drew a lot of attention and engagement. Posts on topic 2 were mostly shared by others (mean 17.83, SD 108.32), suggesting that information on risk reduction and other related health topics was perceived as useful and valuable for sharing with others on their social networks. Topic 2 contained the most negative emotions (mean 4.01, SD 4.25), whereas topics 4 (mean 6.07, SD 5.63) and 5 (mean 6.65, SD 10.00) related to health tips and resource sharing contained the most positive emotions.

A total of 4 topics were identified in the post–COVID-19 comments (Table S9 in Multimedia Appendix 1 shows the topic summaries and examples). Topic 1, unverified information, included advertisements and potential misinformation that promoted unverified physicians and alternative treatments such as herbs. These promotional contents were lengthier than other comments. Topic 2, asking medical questions, was related to inquiries to physicians and cardiologists. Topic 3 was about sharing personal experiences with heart diseases. Topic 4, providing social support, was about people providing social support to each other and discussing risk reduction. Regarding sentiments (Table S10 in Multimedia Appendix 1 shows summary statistics), topic 2 (mean 7.85, SD 9.05) about risk reduction contained the most negative emotions, and topics 3 (mean 28.32, SD 23.99) and 4 (mean 11.03, SD 20.89) related to social support and sharing had the most positive emotions.

In addition, sentiments in posts and comments also changed during the COVID-19 pandemic. Compared with positive (mean 4.55, SD 6.43) and negative (mean 2.70, SD 3.98) emotions before the COVID-19 pandemic, posts became less emotional during the pandemic, with significantly less positive (mean 4.34, SD 5.78; *P*=.002) and negative (mean 2.52, SD 3.41; *P*<.001) emotions. However, in the comments, compared with positive (mean 10.72, SD 19.38) and negative (mean 2.27, SD 6.06) emotions before the COVID-19 pandemic, there were significantly more positive (mean 12.27, SD 19.67; *P*<.001) and negative (mean 3.67, SD 7.46; *P*<.001) emotions during the pandemic.

To summarize, there were specific discussions related to COVID-19, pandemic situations, and risks of heart disease in posts and comments published during the pandemic. The post–COVID-19 topics and comments highlighted the urgency for people to seek web-based information, connect with physicians, and share risk-reduction tips while people were enduring lockdowns, limited health care access, and restricted physical movements and social connection.

### Thematic Topics of Posts on Heart Health for Women

A total of 4 topics were identified in posts about heart health for women (Table S11 in Multimedia Appendix 1 shows the topic summaries and examples). Topic 1, local events for women, was about heart health organizations sharing local events to promote heart health for women and the awareness of women-specific symptoms and prevention. Topic 2, gender-specific symptoms, was information on the differences in heart disease symptoms and warning signs between men and women. Topic 3, sharing information, was about sharing information on specific heart diseases, organs, and surgical procedures. Topic 4, sharing resources, centered on sharing heart health–related resources, including identified misinformation. Table S12 in Multimedia Appendix 1 shows the engagement and sentiment information for the different topics. Posts on topic 3 received the highest number of likes (mean 137.18, SD 322.73), comments (mean 13.15, SD 32.71), and shares (mean 44.63, SD 93.86). This suggests that the public was concerned with the details of cardiovascular diseases and surgical procedures by asking and sharing relevant information and experiences. Posts on topic 1 were the most positive (mean 3.85, SD 4.16), and posts on topic 2 were the most negative (mean 4.87, SD 5.33).

### Thematic Topics of Comments on Heart Health for Women

We extracted 4 topics from comments related to heart health for women (Table S13 in Multimedia Appendix 1 provides topic summaries and examples). Topic 1, sharing symptoms, was about people sharing their own experiences with heart diseases, especially their distinctive warning signs and symptoms that differentiated them from those of men. Topic 2, sharing personal experiences, revolved around survivors of heart diseases sharing experiences after their surgeries and expressing appreciation for their physicians and surgical teams. Topic 3, providing emotional support, was about people providing informational and emotional support for each other by sharing heart health– and heart disease–related information. Topic 4, religious content and support, was about people providing encouragement and thanks and sharing Facebook posts by tagging their Facebook friends in the comments. Table S14 in Multimedia Appendix 1 shows topic engagement and sentiment statistics. Supportive comments on topics 3 (mean 13.49, SD 19.03) and 4 (mean 19.08, SD 29.26) were extremely positive, whereas comments on topic 1 were the most negative (mean 3.85, SD 4.16).

## Discussion

### Principal Findings

This study analyzed heart health– and heart disease–related conversations on Facebook from 2016 to 2021. First, we observed an increase in heart health–related discussions on Facebook from 2016 to 2021. Second, health organizations were major contributors to heart disease and health–related discussions, especially in terms of information dissemination and heart health promotion. Third, the public was concerned about heart health during the COVID-19 pandemic, which was addressed by organizations and physicians. Fourth, we observed an extensive discussion on heart health for women. Finally, we observed some promotional or misleading content on alternative treatments that need to be effectively addressed by health care professionals in the web-based space or the platform. In the following sections, we discuss these findings in more detail.

### Comparison With Prior Work

Social media has become a popular platform for health information exchange, especially for organizations to communicate information related to heart health, promote events, and address the public’s concerns directly on social media [31,32]. From 2016 to 2021, the public’s discussions on heart disease prevention and treatment and the perceived risk of cardiovascular disease increased, indicating a general trend of increased awareness of heart health [33]. Through the theoretical lens of the Health Belief Model, we found that web-based Facebook discussions primarily covered constructs of perceived risks (ie, discussing personal experiences with and opinions on heart diseases), perceived benefits of preventative actions (ie, discussing risk-reduction behaviors), and self-efficacy (ie, discussing prevention and treatment). The fact that organizations and physicians are major contributors to heart health content suggests that Facebook is becoming a useful channel that connects health care professionals and the public and enables health care professionals to deliver useful educational and behavior change messages to the public. The public also leverages the platform to share their own experiences, ask questions, exchange resources, and provide social support, which can potentially contribute to higher collective and individual efficacy in preventing or managing heart diseases [34].

The discussions related to heart health and heart disease on Facebook are mostly contributed to by health organizations such as the American Heart Association. These organizations have used social media to educate the public on heart disease prevention, risk reduction, and treatment [35]. The posts created by health organizations had a positive tone overall, although the posts related to risk reduction were more negative, with warnings of symptoms and negative consequences. In addition, health organizations engaged and interacted with the audience in different ways. Local organizations (eg, state-level organizations) engaged the communities in local events such as hiking to enhance the community’s physical activity, promote heart health knowledge, and build connections with the local community. For example, both topic 1 for all posts (Table 1) and topic 1 for posts on heart health for women (Table S11 in Multimedia Appendix 1) showed the promotion of heart health knowledge and local activities. Health organizations encouraged the audience to share personal experiences with cardiovascular diseases, such as symptoms, treatment, and diagnosis. The audience was responsive by discussing topics in comments similar to topics in posts, such as sharing personal stories and discussing risk-reduction methods. This maintained a healthy community through social interactions and discussions. We want to highlight that health care professionals and physicians directly leverage Facebook to deliver live discussion sessions. This synchronous communication directly connects the public with informational sources where people can exchange questions and concerns in real time [34]. Overall, health organizations contribute significantly to heart health–related discussions on Facebook and promote an interactive and supportive community.

The comparison of the conversations before and during the COVID-19 pandemic informed us of the impact of COVID-19 on individuals with preexisting cardiovascular conditions. Posts during the pandemic specifically focused on risk-reduction practices in diet and exercise as social isolation forced people to live with a different daily routine where securing healthy foods and engaging with sufficient physical activity became very challenging, which posed elevated risks to already vulnerable individuals. Health organizations promptly provided information on COVID-19 and heart health and engaged them in preventive care for heart health during the pandemic [36]. Organizations also addressed the public’s concerns regarding the influence of COVID-19 on heart conditions [36]. The public was responsive to these resources, with high levels of likes, shares, and comments. They also responded to physicians’ live sessions with questions and appreciation. This finding is consistent with previous research showing that people actively seek health information on social media, especially during the COVID-19 pandemic [32].

A prominent conversation was related to heart disease and heart health in women. Women-specific posts accounted for 14.91% (5200/34,885) of all posts. These contents centered on (1) women-specific promotional events as a part of the Go Red for Women campaign to promote the awareness of heart health and heart disease for women and (2) information related to the differences between women and men in warning signs, symptoms, treatments, and prevention. As an old myth goes, heart disease is a “man’s disease” [21]. With the growing promotion of and discussion on heart health for women, such myths have been actively debunked via social media. As social media platforms are preferred channels for women to become informed [37], the public, especially women, may have become more aware of and educated on women-specific symptoms and treatments. In addition to social media content, a study on search queries also supported the increasing awareness of heart health for women [38]. Increasing awareness can help improve the well-being of women and decrease the number of women with cardiovascular diseases.

Finally, we observed a few promotional comments during the pandemic and women-related posts, such as the promotion of alternative treatments for heart disease, cancer, and other major diseases and the specific promotion of physicians with unverified patient narratives and contact information. Although this kind of unverified information accounted for a small portion of the heart health community on Facebook, some individuals may still fall for it. Although our findings generally support the positive role that Facebook has played in promoting public awareness and education on heart health, we still acknowledge that identifying and managing unverified information on the platform is urgently needed as unverified misinformation can affect the public’s health-related attitudes and behaviors. So far, Facebook has not published rules or policies for general or heart health–specific information. A practical route may be for health organizations to maintain their pages or groups to actively monitor and address shared unverified information.

### Limitations

There are a few limitations noted in this research. First, this study focused on Facebook conversations related to heart health. Although it filled a research gap in examining Facebook data, we acknowledge that other social media platforms also support and engage the public on heart health. Data from platforms such as Instagram and Reddit are worth investigating. Second, within the scope of Facebook data, because of platform policies and ethical considerations, we did not obtain data from private groups or comments from public groups. Such data may add more insights into how individual users discuss, relate to, and understand heart diseases in more private web-based interaction settings. Third, we were unable to eliminate the factor of time in the comparison between before and during the COVID-19 pandemic. Although we observed differences in sentiments and thematic topics before and during the COVID-19 pandemic, these differences might not be fully attributable to the COVID-19 pandemic. Finally, this study was observational in nature, and we cannot draw any causal conclusions from this study. Although this study presented public discussions on heart health, we cannot draw any conclusions on how heart health information from organizations may have affected public discussions on heart health.

### Conclusions, Implications, and Future Directions

On the basis of a 5-year data set of public Facebook groups and pages, we observed informative and interactive conversations on Facebook related to heart health and heart disease for the general public, specifically women and individuals with preexisting cardiovascular conditions. The active participation by health organizations, physicians, and the public at both the national and local levels contributed to a diverse discussion with information, resources, experience sharing, and social support.

This study has implications for heart health organizations to engage in two-way communication with the public given the interactive nature of social media platforms [39]. Although posts from organizations are mainly about information and resource sharing, the public still has specific questions regarding heart health and diseases. Posts about physicians’ live sessions received a high volume of attention in terms of the number of likes, comments, and shares. This provides an opportunity for heart health organizations to listen to the audience and address the public’s concerns for more effective health education and promotion [25]. Although we observed an increasing discussion on heart health for women, heart health organizations should provide more gender-specific information for women. Such posts are likely to be further shared among the users’ social networks to benefit other family members and friends who are women [29].

This study provides an overview of heart health discussions on social media, especially in terms of thematic topics and public sentiments. Future studies are needed to analyze heart health discussions on other social media platforms, public forums, and discussion boards to provide a more comprehensive examination of the public discourse on social media. In addition, future studies may investigate how demographic differences play a role in shaping the public discourse on heart health. Disparities in heart health knowledge and health behaviors among different racial and ethnic groups can be examined. We only investigated the distinctive discussions on heart health for women; other demographic characteristics such as age and ethnicity should be further explored. Finally, given the increasing public communication on heart health, studies should be conducted to develop effective communication strategies leveraging social media such as Facebook for more effective health promotion and education.

## Appendix

Multimedia Appendix 1 Supplementary information for study procedure and results.

## Abbreviations

LDA: latent Dirichlet allocation
LIWC: Linguistic Inquiry and Word Count

## Data Availability

Data sharing was not applicable to this study as we used Facebook data that were available to the public and did not generate new data.
