# Supplementary material for: Analyzing Public Conversations About Heart Disease and Heart Health on Facebook From 2016 to 2021: Retrospective Observational Study Applying Latent Dirichlet Allocation Topic Modeling
Source: JMIR Cardio. 2022 Nov 22;6(2):e40764. doi: 10.2196/40764 (PMC9683528; doi:10.2196/40764)
Supplement: Multimedia Appendix 1 [file cardio_v6i2e40764_app1.docx]

# Analyzing Public Conversations of Heart Disease and Heart Health on Facebook from 2016 to 2021: An Observational Study

Supplementary Materials

Table S1. The list of keywords that were used to retrieve Facebook pages, Facebook groups, and Facebook posts.

| Target concepts | Searched terms |
| --- | --- |
| Heart health and disease | heart health, heart disease, heart attack, heart health symptoms, heart disease symptoms, heart attack symptoms, myocardial infarction, blood clots, heart treatment, acute coronary syndrome, coronary artery disease, myocardial infarct, cardiac infarct, angina, cardiac stress |
| Heart health and social support | heart health support, heart disease support, heart attack support |
| Heart health for women | Go Red for Women |

Table S2. Two-sample t-tests comparing sentiments from Linguistic Inquiry and Word Count (LIWC) between Facebook posts (N = 34,885) and comments (N = 51,835).

| LIWC sentiments ^a^ | Posts | | Comments | | P-value |
| --- | --- | --- | --- | --- | --- |
|  | Mean | SD | Mean | SD |  |
| Positive emotion **%** | 4.48 | 6.21 | 11.29 | 19.50 | <.001 |
| Negative emotion **%** | 2.64 | 3.79 | 2.78 | 6.64 | <.001 |
| Anger **%** | 0.87 | 1.91 | 1.06 | 3.50 | <.001 |
| Anxiety **%** | 0.86 | 2.12 | 0.57 | 3.18 | <.001 |
| Sadness **%** | 0.44 | 1.51 | 0.49 | 2.98 | <.001 |

Note. ^a^ Positive and negative emotions represent the percentages of words in a post that fall into either the dictionary of positive emotions or the dictionary of negative emotions.

Table S3. Latent Dirichlet Allocation (LDA) topic modeling for pre-COVID posts (June 2016 - January 1st, 2020), showing topic keywords and weights, topic interpretation, and example posts.

| Topic | Top 10 keywords and weights ^a^ | Interpretation | Example posts (paraphrased) |
| --- | --- | --- | --- |
| 1  Promoting experience sharing | 0.053*heart, 0.015*disease, 0.012*life, 0.011*day, 0.010*family, 0.010*know, 0.009*today, 0.009*time, 0.009*health, 0.009*share | People share personal stories related to heart disease; heart health campaigns encourage people to share to promote awareness | *Grandpa, fathers, and several uncles died of heart attacks. Two brothers had heart attacks. Heart Health is important, I lost 20 lbs from eating and working out everyday. I want to survive!* |
| 2  Sharing local events | 0.040*women, 0.040*heart, 0.037*red, 0.018*free, 0.016*disease, 0.016*thank, 0.015*health, 0.012*american, 0.011*association, 0.011*stroke | Heart health organizations share heart health-related local events to promote heart health for women | *Wisconsin, National Wear Red Day is Friday! #GoRedWearRed to support heart health, and hashtag your city. Help build lives free of cardiovascular diseases and stroke, donate to the American Heart Association.* |
| 3  Risk reduction discussion | 0.054*heart, 0.033*disease, 0.021*risk, 0.016*health, 0.014*blood, 0.013*high, 0.012*pressure, 0.011*stroke, 0.010*cholesterol, 0.010*new | Risk reduction and lifestyle modification for heart health | *Avocados are heart healthy according to the FDA. They reduce bad cholesterol, if they are not in your diet you should add them!* |
| 4  Sharing warning signs | 0.031*heart, 0.029*attack, 0.011*available, 0.010*pain, 0.010*abstract, 0.009*pdf, 0.009*test, 0.009*cardiac, 0.008*chest, 0.007*symptoms | people share information related to warning signs and symptoms of specific heart diseases | *Hypertrophic cardiomyopathy (HCM) is a disease where the heart muscle becomes very thick, which makes it harder to pump blood to your body.* |
| 5  Doctors’ live discussion sessions | 0.037*dr, 0.020*heart, 0.015*page, 0.015*th, 0.014*attack, 0.012*pm, 0.011*beautiful, 0.011*thankful, 0.011*cardio, 0.011*hospital | Facebook live sessions of doctors sharing of heart health-related info | *Facebook LIVE session where Dr. S discusses lifestyle management after a heart attack.* |

Note. ^a^ Asterisk (*) shows the weight of each keyword.

Table S4. Latent Dirichlet Allocation (LDA) topic modeling for pre-COVID posts (June 2016 - January 1st, 2020): topic distribution, Facebook metrics, and sentiments from Linguistic Inquiry and Word Count (LIWC).

| Topic | Interpretation | #posts **%** | Facebook metrics ^a^ | | | Sentiments from LIWC ^b^ | | |
| --- | --- | --- | --- | --- | --- | --- | --- | --- |
| Topic |  |  | #likes  M (SD) | #comments  M (SD) | #shares  M (SD) | Word count  M (SD) | Positive emotion **%**  M (SD) | Negative emotion **%**  M (SD) |
| 1  Promoting experience sharing | People share personal stories related to heart disease; heart health campaigns encourage people to share to promote awareness | 6534 (29%) | 52.74 (281.13) | 6.26 (23.86) | 15.44 (84.56) | 51.37 (60.26) | 5.61 (5.78) | 2.12 (3.36) |
| 2  Sharing local events | Heart health organizations share heart health-related local events to promote heart health for women | 5709 (25.3%) | 41.51 (237.98) | 2.39 (15.20) | 14.88 (124.32) | 45.76 (34.26) | 4.90 (4.90) | 1.10 (2.38) |
| 3  Risk reduction discussion | Risk reduction and lifestyle modification for heart health | 6125 (27.2%) | 61.38 (243.13) | 2.64 (13.10) | 21.33 (103.09) | 38.29 (38.39) | 3.21 (4.48) | 4.28 (4.59) |
| 4  Sharing warning signs | people share information related to warning signs and symptoms of specific heart diseases | 3088 (13.7%) | 70.99 (264.09) | 8.87 (25.58) | 36.73 (174.07) | 62.69 (91.68) | 5.25 (11.54) | 3.85 (5.00) |
| 5  Doctors’ live discussion sessions | Facebook live sessions of doctors sharing of heart health-related info | 1089 (4.8%) | 55.96 (150.94) | 11.01 (31.19) | 13.78 (44.22) | 46.19 (34.25) | 1.98 (3.49) | 2.51 (2.65) |

Notes. ^a^ Data collected in November 2021.

^b^ Positive and negative emotions represent the percentages of words in a post that fall into either the dictionary of positive emotions or the dictionary of negative emotions.

Table S5. Latent Dirichlet Allocation (LDA) topic modeling for pre-COVID comments (June 2016 - January 1st, 2020): topic keywords and weights, topic interpretation, and example comments.

| Topic | Top 10 keywords and weights ^a^ | Interpretation | Example comments (paraphrased) |
| --- | --- | --- | --- |
| 1  Sharing warning signs | 0.070*heart, 0.026*attack, 0.018*years, 0.011*surgery, 0.010*ago, 0.009*valve, 0.009*pain, 0.009*blood, 0.008*stents, 0.008*disease | People share warning signs and symptoms of heart diseases and discuss doctors' diagnoses | *Heart attack signs are different for women. It is the leading cause of death for women in the US, yet many women write the symptoms off as other minor things. Take it seriously.* |
| 2  Sharing risk reductions | 0.016*good, 0.014*god, 0.014*time, 0.013*go, 0.013*work, 0.012*know, 0.010*like, 0.010*feel, 0.010*help, 0.010*day | People share the relationship aspect of heart health and risk reductions | *Everyday I walk and I lift weights 2-3 days a week. I might give up the gym because it completely exhausts me these days. After 3 years of trying, I still get very tired.* |
| 3  Providing emotional support | 0.037*thank, 0.025*heart, 0.021*love, 0.017*share, 0.014*family, 0.013*disease, 0.011*amaze, 0.011*story, 0.009*health, 0.009*prayers | People provide emotional support and suggestions on risk reductions to others | *Thank you A for the support of the community. We are trying to help underserved patients in the world. It’s amazing to see the faces of those helped by this work.* |
| 4  Religious contents | 0.039*great, 0.027*thank, 0.019*dr, 0.014*good, 0.014*awesome, 0.014*true, 0.013*amen, 0.012*information, 0.012*love, 0.011*nice | Religious content: thank to god and doctors | *They saved my husband’s life. Grateful forever for the doctors and the whole team.* |
| 5  General health discussions | 0.026*red, 0.020*smoke, 0.015*wear, 0.014*congratulations, 0.010*quit, 0.008*drink, 0.007*happy, 0.007*day, 0.006*cause, 0.005*interest | Discussions on other health-related topics (e-cigarettes) | *I was a teen and wanted to smoke so I did. The flavour didn’t matter. Kids will smoke or vape if they want, banning flavours will not stop them!*  *Another scare tactic by anti groups. Hey… Go ahead and smoke cigs!* |

Note. ^a^ Asterisk (*) shows the weight of each keyword.

Table S6. Latent Dirichlet Allocation (LDA) topic modeling for pre-COVID comments (June 2016 - January 1st, 2020): topic distribution, Facebook metrics, and sentiments from Linguistic Inquiry and Word Count (LIWC).

| Topic | Interpretation | Facebook metrics ^a^ | Sentiments from LIWC ^b^ | | |
| --- | --- | --- | --- | --- | --- |
|  |  | #comments **%** | Word count  M (SD) | Positive emotion **%**  M (SD) | Negative emotion **%**  M (SD) |
| 1  Sharing warning signs | People share warning signs and symptoms of heart diseases and discuss doctors' diagnoses | 10097 (30.8%) | 28.84 (35.82) | 2.37 (4.74) | 4.01 (6.35) |
| 2  Sharing risk reductions | People share the relationship aspect of heart health and risk reductions | 7363 (22.5%) | 25.00 (40.08) | 9.36 (16.18) | 2.49 (7.16) |
| 3  Providing emotional support | People provide emotional support and suggestions on risk reductions to others | 5912 (18.0%) | 15.41 (27.61) | 16.64 (22.77) | 1.17 (4.45) |
| 4  Religious contents | Religious content: thank to god and doctors | 5460 (16.7%) | 7.70 (10.73) | 23.17 (27.18) | 0.77 (5.12) |
| 5  General health discussions | Discussions on other health-related topics (e-cigarettes) | 3942 (12.0%) | 10.51 (25.78) | 8.51 (17.89) | 1.14 (5.13) |

Notes. ^a^ Data collected in November 2021.

^b^ Positive and negative emotions represent the percentages of words in a post that fall into either the dictionary of positive emotions or the dictionary of negative emotions.

Table S7. Latent Dirichlet Allocation (LDA) topic modeling for post-COVID posts (January 1st, 2020 - June 2021): topic keywords and weights, topic interpretation, and example posts.

| Topic | Top 10 keywords and weights ^a^ | Interpretation | Example posts (paraphrased) |
| --- | --- | --- | --- |
| 1  Doctors’ live discussion sessions | 0.043*article, 0.039*video, 0.020*content, 0.017*information, 0.016*dr, 0.015*presentation, 0.015*attack, 0.012*health, 0.012*heart, 0.012*advice | Facebook live sessions of doctors sharing of heart health-related info | *Dr. J, a consulting physician, discusses how to prevent Heart Attack* |
| 2  Risk reduction discussions | 0.055*heart, 0.028*attack, 0.026*disease, 0.012*know, 0.010*risk, 0.008*angina, 0.008*patients, 0.007*doctor, 0.007*cause, 0.007*prinzmetal | Organizations provide health tips on risk reductions and other topics related to heart health, such as flu shot safety and related diseases | *Those who have heart disease have a 10 times higher chance of heart attack within 3 days of contracting the flu.*  *C didn’t trust the HA symptoms for 8 weeks. [C] wants others to know better. COVID19 still has hospitals as the safest place to have medical emergencies. Don’t die of doubt.* |
| 3  Risk reduction discussions for women | 0.045*heart, 0.037*red, 0.035*women, 0.022*disease, 0.015*awareness, 0.013*join, 0.012*thank, 0.011*health, 0.010*support, 0.009*wear | Provide health tips on risk reduction and organize local campaigns to promote heart health for women | *The no1 killer of women is cardiovascular disease. Raise awareness and funds to change that, join us for the GO Red for Women Wellness Retreat!* |
| 4  Risk reduction discussions for the pandemic | 0.039*heart, 0.018*health, 0.012*help, 0.012*free, 0.011*life, 0.011*day, 0.010*healthy, 0.010*feel, 0.009*years, 0.008*time | Provide health tips on risk reduction for heart health in pandemic | *Best Friend Fridays are coming back! Bring your pet into your workday as pet owners shift routines during the pandemic.* |
| 5  Resources sharing | 0.038*blood, 0.026*pressure, 0.016*high, 0.015*pdf, 0.012*cholesterol, 0.008*body, 0.007*thing, 0.007*eat, 0.007*level, 0.007*gtn | Share resources related to heart health | *Cholesterol is a substance your liver produces that is vital for many functions, but also dangerous. Read more:* |

Note. ^a^ Asterisk (*) shows the weight of each keyword.

Table S8. Latent Dirichlet Allocation (LDA) topic modeling for post-COVID posts (January 1st, 2020 - June 2021): topic distribution, Facebook metrics, and sentiments from Linguistic Inquiry and Word Count (LIWC).

| Topic | Interpretation | #posts **%** | Facebook metrics ^a^ | | | Sentiments from LIWC ^b^ | | |
| --- | --- | --- | --- | --- | --- | --- | --- | --- |
|  |  |  | #likes  M (SD) | #comments  M (SD) | #shares  M (SD) | Word count  M (SD) | Positive emotion **%**  M (SD) | Negative emotion **%**  M (SD) |
| 1  Doctors’ live discussion sessions | Facebook live sessions of doctors sharing of heart health-related info | 1262 (10.2%) | 61.55 (112.25) | 10.08 (18.12) | 5.67 (17.95) | 134.82 (42.75) | 2.74 (6.22) | 2.8 (1.01) |
| 2  Risk reduction discussions | Organizations provide health tips on risk reductions and other topics related to heart health, such as flu shot safety and related diseases | 3636 (29.5%) | 54.77 (256.26) | 6.22 (29.97) | 17.83 (108.32) | 55.11 (75.48) | 2.33 (3.34) | 4.01 (4.25) |
| 3  Risk reduction discussions for women | Provide health tips on risk reduction and organize local campaigns to promote heart health for women | 3700 (30.0%) | 32.84 (241.42) | 2.8 (19.45) | 9.27 (99.42) | 45.71 (30.89) | 4.96 (5.18) | 1.22 (2.2) |
| 4  Risk reduction discussions for the pandemic | Provide health tips on risk reduction for heart health in pandemic | 2679 (21.7%) | 24.59 (97.81) | 3.73 (16.74) | 6.38 (34.29) | 52.48 (70.02) | 6.07 (5.63) | 2.11 (3.16) |
| 5  Resources sharing | Share resources related to heart health | 1063 (8.6%) | 11.81 (55.08) | 1.13 (4.69) | 4.79 (29.99) | 49.73 (62.68) | 6.65 (10) | 2.65 (4.01) |

Notes. ^a^ Data collected in November 2021.

^b^ Positive and negative emotions represent the percentages of words in a post that fall into either the dictionary of positive emotions or the dictionary of negative emotions.

Table S9. Latent Dirichlet Allocation (LDA) topic modeling for post-covid comments (January 1st, 2020 - June 2021): topic keywords and weights, topic interpretation, and example comments.

| Topic | Top 10 keywords and weights ^a^ | Interpretation | Example comments (paraphrased) |
| --- | --- | --- | --- |
| 1  Misinformation | 0.011*need, 0.010*go, 0.009*get, 0.009*years, 0.008*know, 0.008*time, 0.007*take, 0.007*blood, 0.007*dr, 0.007*tell | Misinformation on alternative treatments and promote doctors | *I’m [W], from the USA. I had HIV in 2011, I saw a new doctor who sent me herbal medicine to cure my HIV with no side effects, he also cures ALS, Hep B, Cancer, Herpes and more! Call him at XXX.* |
| 2  Asking medical questions | 0.203*heart, 0.117*attack, 0.074*sir, 0.034*good, 0.015*prayers, 0.015*disease, 0.014*cause, 0.010*risk, 0.009*even, 0.009*symptoms | People ask doctors about heart diseases and risk reduction | *Does running when young help heart health even if not running when older?*  *How much is a patient in danger of COVID if they have diabetes and cardiac complications?* |
| 3  Sharing personal experiences | 0.057*thank, 0.051*valve, 0.032*information, 0.024*great, 0.024*sir, 0.022*year, 0.021*happy, 0.018*new, 0.016*heart, 0.016*nice | people share personal experiences with heart disease treatments | *Born with a heart murmur, ok until 2018. Had aortic valve replaced at 35, along with an aortic aneurysm. They discovered I had a unicuspid valve, not bicuspid.*  *My husband had a mitral replaced with a pig valve, which lasted 21 years. New valve is from a cow.* |
| 4  Providing emotional support | 0.017*patients, 0.016*love, 0.012*take, 0.009*yes, 0.008*week, 0.008*blood, 0.007*congratulations, 0.007*certain, 0.007*wear, 0.007*quantity | Emotional support and discussions on risk reduction | *Dr. D, Dr A, Dr A, and staff… Amazing!* |

Note. ^a^ Asterisk (*) shows the weight of each keyword.

Table S10. Latent Dirichlet Allocation (LDA) topic modeling for post-covid comments (January 1st, 2020 - June 2021): topic distribution, Facebook metrics, and sentiments from Linguistic Inquiry and Word Count (LIWC).

| Topic | Interpretation | Facebook metrics ^a^ | Sentiments from LIWC ^b^ | | |
| --- | --- | --- | --- | --- | --- |
|  |  | #comments **%** | Word count  M (SD) | Positive emotion **%**  M (SD) | Negative emotion **%**  M (SD) |
| 1  Misinformation | Misinformation on alternative treatments and promote doctors | 6361 (33.4%) | 26.44 (36.97) | 5.64 (11.44) | 3.44 (7.49) |
| 2  Asking medical questions | People ask doctors about heart diseases and risk reduction | 5401 (28.3%) | 8.08 (6.18) | 7.77 (15.10) | 7.85 (9.05) |
| 3  Sharing personal experiences | People share personal experiences with heart disease treatments | 4366 (22.9%) | 8.50 (10.84) | 28.32 (23.99) | 0.30 (1.84) |
| 4  Providing emotional support | Emotional support and discussions on risk reduction | 2933 (15.4%) | 9.63 (18.34) | 11.03 (20.89) | 1.47 (5.47) |

Notes. ^a^ Data collected in November 2021.

^b^ Positive and negative emotions represent the percentages of words in a post that fall into either the dictionary of positive emotions or the dictionary of negative emotions.

Table S11. Latent Dirichlet Allocation (LDA) topic modeling for posts related to heart health for women: topic keywords and weights, topic interpretation, and example posts.

| Topic | Top 10 keywords and weights ^a^ | Interpretation | Example posts (paraphrased) |
| --- | --- | --- | --- |
| 1  Local events about women | 0.063*red, 0.052*women, 0.041*heart, 0.027*disease, 0.023*wear, 0.016*day, 0.015*awareness, 0.012*national, 0.010*today, 0.010*stroke | Heart health organizations share heart health-related local events to promote heart health for women | *Wear Red on National Wear Red day. 1 in 3 deaths among women are from heart disease and stroke. 80% of cardiac and stroke events can be prevented, which is why we encourage wearing red for awareness of others.* |
| 2  Gender-specific symptoms | 0.089*heart, 0.040*disease, 0.036*attack, 0.028*women, 0.017*know, 0.017*symptoms, 0.014*sign, 0.011*men, 0.011*pain, 0.009*learn | The differences of warning signs and symptoms of heart disease between men and women | *International Women’s day is today. Share this info on women's health! Warning signs for heart attack are different between men and women.* |
| 3  Sharing information | 0.025*heart, 0.016*coronary, 0.014*patients, 0.010*disease, 0.010*blood, 0.010*artery, 0.010*treatment, 0.010*angina, 0.007*valve, 0.007*atrial | Information sharing related to specific heart diseases, organs, and surgery procedures | *Dr. C is telling us about the watchman procedure.*  *The largest artery in the body is the aorta. It carries blood with oxygen from the heart to the body.* |
| 4  Sharing resources | 0.106*available, 0.099*abstract, 0.010*peripheral, 0.010*pad, 0.009*dr, 0.008*vein, 0.007*artery, 0.006*vascular, 0.006*defect, 0.005*legs | People share resources related to heart health; there are misinformation on alternative treatments as well | *Manuscript for the above article: one page available only.* |

Note. ^a^ Asterisk (*) shows the weight of each keyword.

Table S12. Latent Dirichlet Allocation (LDA) topic modeling for posts related to heart health for women: topic distribution, Facebook metrics, and sentiments from Linguistic Inquiry and Word Count (LIWC).

| Topic | Interpretation | #posts **%** | Facebook metrics ^a^ | | | Sentiments from LIWC ^b^ | | |
| --- | --- | --- | --- | --- | --- | --- | --- | --- |
|  |  |  | #likes  M (SD) | #comments  M (SD) | #shares  M (SD) | Word count  M (SD) | Positive emotion **%**  M (SD) | Negative emotion **%**  M (SD) |
| 1  Local events about women | Heart health organizations share heart health-related local events to promote heart health for women | 2117 (40.7%) | 45.85 (180.48) | 4.06 (25.57) | 29.64 (198.12) | 36.88 (24.44) | 3.85 (4.16) | 1.33 (2.32) |
| 2  Gender-specific symptoms | The differences of warning signs and symptoms of heart disease between men and women | 1719 (33.1%) | 73.5 (346.01) | 4.62 (19.65) | 39.46 (172.64) | 40.96 (41.76) | 1.7 (2.55) | 4.87 (5.33) |
| 3  Sharing information | Information sharing related to specific heart diseases, organs, and surgery procedures | 851 (16.4%) | 137.18 (322.73) | 13.15 (32.71) | 44.63 (93.86) | 84.76 (97.76) | 1.81 (2.47) | 2.38 (2.79) |
| 4  Sharing resources | People share resources related to heart health; there are misinformation on alternative treatments as well | 513 (9.9%) | 32.8 (230.06) | 2.24 (11.66) | 9.52 (47.09) | 20.11 (38.45) | 1.06 (3.64) | 0.77 (2.69) |

Notes. ^a^ Data collected in November 2021.

^b^ Positive and negative emotions represent the percentages of words in a post that fall into either the dictionary of positive emotions or the dictionary of negative emotions.

Table S13. Latent Dirichlet Allocation (LDA) topic modeling for comments related to heart health for women: topic keywords and weights, topic interpretation, and example comments.

| Topic | Top 10 keywords and weights ^a^ | Interpretation | Example comments (paraphrased) |
| --- | --- | --- | --- |
| 1  Sharing symptoms | 0.041*heart, 0.020*attack, 0.014*go, 0.014*pain, 0.013*years, 0.010*time, 0.010*feel, 0.009*tell, 0.009*say, 0.009*get | People share personal experiences with heart disease and the differences of warning signs and symptoms of heart disease between men and women | *Mom had a heart attack at 63, was tired and had jaw pain. I didn’t even have chest pain, throat, pulled muscle, felt off a bit.* |
| 2  Sharing personal experiences | 0.039*heart, 0.034*valve, 0.027*red, 0.019*wear, 0.013*surgery, 0.012*aortic, 0.012*dr, 0.012*great, 0.010*mitral, 0.010*years | People share personal experiences with surgeries and express thanks to doctors | *Awesome heart hospital! Dr L. was my mothers doctor for 10 years before my mother passed at 90. We loved Dr. L!*  *Three heart surgeries, 2014 valve replacement, 2017 open heart valve repair, 2019 open heart valve replacement. Thank God for every day.* |
| 3  Providing emotional support | 0.038*thank, 0.025*heart, 0.019*women, 0.018*disease, 0.013*share, 0.011*know, 0.010*good, 0.008*story, 0.008*life, 0.008*help | People provide emotional support and heart health-related information with others; there are misinformation as well | *Thanks for the story! K is the co-founder of SCAD Alliance and advocates for SCAD research, education, and support. She is an asset!*  *Healthy as possible is the goal. Many who were exercising and fit still had symptoms, and its hard to continue the lifestyle. Wish those without normal risk factors would be studied more.* |
| 4  Religious contents and support | 0.026*bless, 0.024*god, 0.013*true, 0.012*awesome, 0.011*false, 0.011*congratulations, 0.010*love, 0.009*yes, 0.008*amen, 0.007*wow | People provide emotional support to others and share posts with their Facebook friends by tagging them in the comments | *God bless you, be strong. Remember how awesome God is. I have good cardiologists, talk to me.* |

Note. ^a^ Asterisk (*) shows the weight of each keyword.

Table S14. Latent Dirichlet Allocation (LDA) topic modeling for comments related to heart health for women: topic distribution, Facebook metrics, and sentiments from Linguistic Inquiry and Word Count (LIWC).

| Topics | Interpretation | Facebook metrics ^a^ | Sentiments from LIWC ^b^ | | |
| --- | --- | --- | --- | --- | --- |
|  |  | #comments **%** | Word count  M (SD) | Positive emotion **%**  M (SD) | Negative emotion **%**  M (SD) |
| 1  Sharing symptoms | People share personal experiences with heart disease and the differences of warning signs and symptoms of heart disease between men and women | 3198 (33.7%) | 34.31 (47.39) | 2.53 (5.6) | 3.09 (5.48) |
| 2  Sharing personal experiences | People share personal experiences with surgeries and express thanks to doctors | 2393 (25.2%) | 18.26 (21.64) | 8.58 (15.78) | 0.97 (2.93) |
| 3  Providing emotional support | People provide emotional support and heart health-related information with others; there are misinformation as well | 2283 (24%) | 15.51 (23.89) | 13.49 (19.03) | 1.75 (5.71) |
| 4  Religious contents and support | People provide emotional support to others and share posts with their Facebook friends by tagging them in the comments | 1627 (17.1%) | 7.9 (11.42) | 19.08 (29.26) | 0.93 (5.56) |

Notes. ^a^ Data collected in November 2021.

^b^ Positive and negative emotions represent the percentages of words in a post that fall into either the dictionary of positive emotions or the dictionary of negative emotions.

Table S15. The number of Facebook posts and comments in the first half (January 1st to June 30th) and second half (July 1st to December 31st) of a year from 2017 to 2020, and the percentage of the number of posts/comments in the first half of a year in all posts/comments of that year.

|  | Facebook posts | | | Facebook comments | | |
| --- | --- | --- | --- | --- | --- | --- |
|  | Jan - Jun | Jul - Dec | % Jan - Jun | Jan - Jun | Jul - Dec | % Jan - Jun |
| 2017 | 2932 | 2874 | 50.5% | 3949 | 4622 | 46.1% |
| 2018 | 2646 | 3592 | 42.4% | 3196 | 5639 | 36.2% |
| 2019 | 3792 | 3607 | 51.3% | 6464 | 5036 | 56.2% |
| 2020 | 3422 | 5128 | 40.0% | 4200 | 10417 | 28.7% |
